# Phylogeny-driven target selection for large-scale genome-sequencing (and other) projects

**DOI:** 10.4056/sigs.3446951

**Published:** 2013-05-20

**Authors:** Markus Göker, Hans-Peter Klenk

**Affiliations:** 1 Leibniz Institute DSMZ – German Collection of Microorganisms and Cell Cultures, Braunschweig, Germany

**Keywords:** phylogenetic diversity, genomics, taxon selection, 16S rRNA, tree of life, Genomic Encyclopedia, *Roseobacter* clade

## Abstract

Despite the steadily decreasing costs of genome sequencing, prioritizing organisms for sequencing remains important in large-scale projects. Phylogeny-based selection is of interest to identify those organisms whose genomes can be expected to differ most from those that have already been sequenced. Here, we describe a method that infers a phylogenetic scoring independent of which set of organisms has previously been targeted, which is computationally simple and easy to apply in practice. The scoring itself, as well as pre- and post-processing of the data, is illustrated using two real-world examples in which the method has already been applied for selecting targets for genome sequencing. These projects are the JGI CSP Genomic Encyclopedia of *Bacteria* and *Archaea* phase I, targeting 1,000 type strains, and, on a smaller-scale, the phylogenomics of the *Roseobacter* clade. Potential artifacts of the method are discussed and compared to a selection approach based on the taxonomic classification.

## Introduction

The Genomic Encyclopedia of *Bacteria* and *Archaea* (GEBA) project was established as a collaboration between the DOE Joint Genome Institute (JGI, Walnut Creek, CA) and a Biological Resource Center (BRC), the German Collection of Microorganisms and Cell Cultures (DSMZ). The goal of GEBA is to obtain reference genomes that more broadly cover the evolutionary diversity of prokaryotes. Once sequencing and annotation are completed, GEBA genomes are submitted to the INSDC databases and made available to the public in the Integrated Microbial Genomes system [1]. The genome sequences are provided together with metadata in a standards-compliant way [2].

GEBA focuses on cultured isolates that have a formal species description (type strains). A frequent misconception is that the types used in taxonomy (type strains, type species, type genera etc.) are *taxonomic* types used for representing a certain taxon by its most typical member. If so, they were bound to, and dependent on, certain taxonomic views such as species concepts or even the general notion that evolution is best represented by a hierarchical classification such as the currently dominating Linnean taxonomy [3]. The critique of hierarchical classifications as being unsuitable for microbiology because of the occurrence of lateral gene transfer, yielding rather a network than a hierarchy [4], would then also affect GEBA. But types are *nomenclatural* constructs which, given a certain taxonomic view, define which names are to be used for a taxon [5]. In microbiology, the use of type strains for genome projects has the additional practical advantage that these strains are guaranteed, or nearly so, to be deposited in at least two distinct culture collections in two distinct countries [6,7]. This ensures that living material is available for follow-up studies that test genome-sequence-derived hypotheses. The availability of biological reference material or even genomic DNA (gDNA) [8] is a great step forward to ensuring reproducibility of the results [2].

The target organisms of GEBA are selected using a 16S rRNA gene-sequence-based phylogenetic tree (the gene on which the current bacterial and archaeal classification is largely based [6,9]), progressively filling in the genomic gaps [10]. Phylogeny-driven genome-sequencing projects are promising for improving microbial classification [4] and particularly for the binning of metagenomic sequences [10]. In the long term, the genomes of representatives of each branch of the tree of life, and of all type strains at the time of accession into public culture collections, will likely be sequenced. But GEBA targeted the organisms deemed genomically more interesting [10] first, and thus required a phylogeny-derived scoring system [11,12] covering all strains of potential interest.

GEBA started with a pilot project (165 strains) that was subsequently extended to approximately 250 target strains and then followed by two phases of 1,000 target strains each. About 140 GEBA genomes have been published at the time of writing (October 2012). For instance, target organisms of the GEBA pilot project included the type strains of *Ktedonobacter racemifer*, the bacterium with the largest genome sequence obtained to date [13], and *Pyrolobus fumarii*, the archaeon with the highest known optimal temperature [14]. Taxonomic conclusions (e.g., reclassifications) were drawn from some of the newly obtained genomic information [15,16].

Here, we describe the design goals and implementation of the phylogeny-based scoring system used for selecting the targets of GEBA phases I and II, which aim to sequence an additional 1,000 microbial type-strain genomes, each. Some examples are provided to illustrate the results for the GEBA project itself and for a more concise project that targets a much smaller group of organisms, the *Roseobacter* clade [17,18] within *Rhodobacteraceae* (*Alphaproteobacteria*) [19].

## Material and methods

### Design goals of the phylogenetic scoring

The major goals of the novel approach were that the scoring (i) is independent of changes in the set of ongoing or finished genome projects, (ii) considers the contribution of a species to the total phylogenetic diversity, as measured using branch lengths, (iii) gives a relatively low weight to organisms in densely sampled groups and a relatively high weight to isolated species, and (iv) if summed up over all leaves of a subtree would provide a biologically sensible score for this subtree. The first goal, independence of changes in the set of ongoing or finished genome projects, was primarily of practical importance, to avoid recalculation of the scores each time a genome project is initialized. A stable score that only depends on the underlying phylogenetic tree is also much easier to use for calculating summary statistics; examples are given below. Further, the same scores can be used for distinct projects if the scoring depends only on a phylogenetic hypothesis, but not on the set of (un-)selected targets. In addition to genome sequencing, phylogeny-based target selection might indeed be of interest in projects on the extraction of secondary metabolites such as antibiotics (e.g., [20-25]), pigments [26] or siderophores [27]. Genome sequencing of phylogenetically selected strains revealed more novel protein families than sequencing randomly selected targets [10]. Hence, it is promising to apply phylogeny-based target selection also to phenotypic investigations, as phylogenetically more distant organisms might be expected to display more divergent phenotypes than close relatives.

The second goal, to consider the contribution of a species to the total phylogenetic diversity in the scoring, as measured using branch lengths [10], is justified as follows. Whereas a rooted tree topology alone indicates the relative branching order, the lengths of the branches also indicate the expected or minimal number of character changes on the respective branch [28], depending on whether the tree was estimated under maximum likelihood [29] or maximum parsimony [30]. These character changes within the dataset (e.g., gene) from which the tree has been inferred can then serve as a proxy for the estimated number of changes within the characters of interest (e.g., content of protein families [10] and possibly also selected phenotypic traits, see above). This approach apparently only presupposes that some correlation exists between the rates of change of the distinct kinds of characters looked at, but it does not presuppose the existence of a molecular (or even phenotypic) clock [28].

For two reasons, another design goal was to weight the score of species in densely sampled groups of organisms downwards and to weight the score of relatively isolated species upwards. First, in the course of the GEBA pilot project the problem sometimes occurred that comparatively closely related organisms were targeted. Second, it is more likely for a more densely sampled group of organisms that a genome of at least one of its members will be targeted by a genome project other than GEBA than for an isolated organism or group of organisms.

The final goal of the novel algorithm was that the score, if summed up over all leaves (i.e., species or subspecies present; see below) of the underlying phylogenetic tree, yielded a value that served as the score of the entire tree in some biologically sensible manner. This feature allowed for estimates of the number of genome projects needed to cover a certain percentage of the total phylogenetic diversity. If phylogenetic diversity was measured using a sum-of-branch-length approach, it should be possible to simply add the scores of distinct subtrees, including the scores of distinct leaves, together to obtain the scores of their parent subtrees or the entire underlying phylogenetic hypothesis. With such an approach, it would be easily possible to assess saturation effects caused by the inclusion of suitable targets.

## Algorithm

We devised a scoring system for the leaves in a rooted topology with branch lengths. To comply with the second design goal, it was obvious that the branch lengths between each leaf and the root node had to be added up in some manner. To agree with the first design goal, this had to be done irrespective of whether any leaves were already marked in some way (e.g., as already targeted for genome projects). That is, none of the leaves themselves could be downweighted or even deleted. For compliance with the fourth design goal, however, some downweighting had to be applied to avoid counting branches several times, thus overestimating overall phylogenetic diversity. For this reason, we considered scores, henceforth called Relative Phylogenetic Diversity (RPD), which proportionally downweighted the lengths of shared (i.e., internal) branches. Two versions were examined, a balanced (bRPD) and an unbalanced (uRPD) version. The latter weights each pair of sister clades equally, irrespective of the respective number of leaves, whereas bRPD takes the subtree sizes into account. Probabilistic interpretations come into play here.

**Figure 1:**
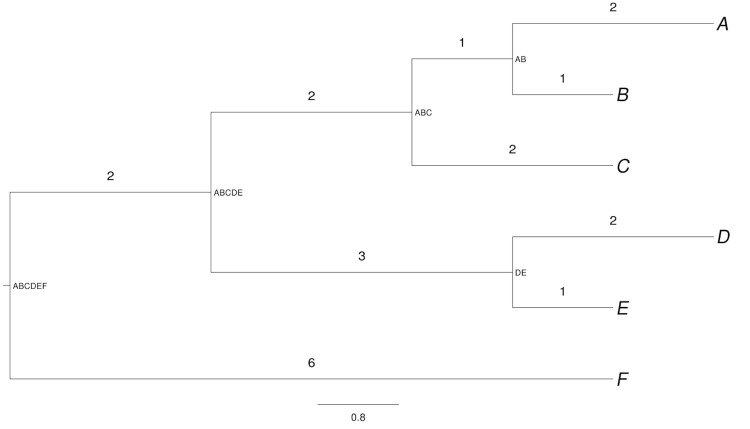
Hypothetical example phylogeny. The numbers above the branches indicate the branch lengths; internal edge labels derived from the names of the leaves of the corresponding subtrees have been added to ease the navigation.

For example, consider leaf *A* in Figure 1. The branch between nodes *A* and *AB* is not shared with another leaf; character changes that occurred on it (whose amount is proportional to the branch length) may have led to, e.g., novel sets of proteins in *A* [10], but not in any other leaf. Changes on the branch between nodes *AB* and *ABC*, however, have affected both *A* and *B*, whereas those on the branch between *ABC* and *ABCDE* have influenced the leaves *A*, *B* and *C*. Proportional weighting thus yields bRPD(*A*) = 2/1 + 1/2 + 2/3 + 2/5 = 3.567. 

Let *N_j_* be the number of branches (edges) between leaf *j* and the root, *b_ij_* be length of the *i*-th one (counted downwards, from leaf to root) of these branches and *s_ij_* be the total number of leaves of the subtree defined by this branch. bRPD then becomes


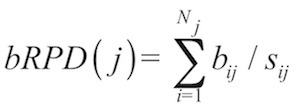


whereas uRPD is defined as


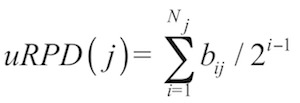


This kind of weighting yields, for example, uRPD(*A*) = 2/1 + 1/2 + 2/4 + 2/8 = 3.25 (Table 1). uRPD apparently only makes sense in strictly dichotomous trees (such as the best-known maximum-likelihood tree of a certain dataset; see below). If bRPD is summed up over all leaves, each branch will be counted exactly as many times as it has leaves. For this reason, the overall bRPD sum is equal to the overall sum of branch lengths of the tree. Whereas the weighting of each branch can differ between its distinct leaves in the case of uRPD, the denominator of formula (2), if averaged over all leaves of a branch, becomes equal to one divided by the number of these leaves, as could easily be proven by complete induction. Hence, if uRPD summed up over all leaves yields the same number as bRPD, the sum of the lengths of all branches of the tree.

**Table 1:** Phylogenetic diversity metrics for the leaves of the example tree in Figure 1.^†^

**Leaf**	**Height**	**bRPD**	**uRPD**	**# nodes**
**A**	7.000	3.567	3.250	4
**B**	6.000	2.567	2.250	4
**C**	6.000	3.067	3.500	3
**D**	7.000	3.900	4.000	3
**E**	6.000	2.900	3.000	3
**F**	6.000	6.000	6.000	1

^†^For each leaf, the distance to the root as obtained by adding up the lengths of all branches between the leaf and the root (“Height”), the balanced Relative Phylogenetic Diversity (“bRPD”), the unbalanced Relative Phylogenetic Diversity (“bRPD”) and the number of nodes between the leaf and the root (including the leaf; “# nodes”) are given.

We conclude that both weighting regimes comply with three of the four design goals listed above. The formulas and the example also indicate that topologically more isolated organisms receive higher scores. The relevant branch lengths of leaves located in less densely populated subtrees will be less severely downweighted. For instance, in Figure 1 *A* and *D* have the same sum-of-branch length distance to the root (7.0), but *D* is topologically more isolated (three instead of four nodes between leaf and root) and, as a consequence, receives a higher score.

The scoring algorithm was implemented as a recursive method using code from the BioRuby library [31] for parsing Newick files and representing trees.

### Selection of a gene and a phylogenetic tree

It is generally agreed upon that, other things being equal, sampling of more characters yields more accurate phylogenies [28]. This is the major reason why genome-sequencing projects are so promising for the purpose of developing a natural classification [4]. Target selection for genome sequencing, however, apparently cannot rely on genome-scale data because these are the very data that will only be generated in the course of the respective project [10]. For this reason, a comprehensive sampling of taxa, not of characters, is crucial for target selection not to overlook promising candidates. The only comprehensively sampled gene for *Archaea* and *Bacteria*, however, is the 16S rRNA gene [9], as in current practice in microbial taxonomy every description of a novel species is accompanied by a newly generated sequence of this gene [6].We chose the most recent version the Living Tree Project (LTP) [32] as underlying phylogenetic hypothesis. The LTP infers a maximum-likelihood phylogeny from a 16S rRNA gene alignment of quality-checked sequences constructed with tools compatible with ARB [33]. Collaborations with a number of BRCs ensured a rather comprehensive sampling. The tree is delivered with branch lengths in Newick format and rooted at the *Archaea*-*Bacteria* split [34]. During the planning phase of the GEBA main project, the last available LTP version (release LTPs102) was from September 2010, comprising 8,029 leaves (and almost as many species, as some were represented by several subspecies). We also calculated the phylogenetic-diversity scores from the LTPs106 release (contained 8,815 leaves) to assess the stability of the results with respect to taxon sampling.

### Detection of ongoing or finished genome projects

While the scoring was designed as independent of the distribution of genome projects (see above), it was necessary to figure out whether organisms with promising genome sequences – according to their score – had already been targeted by a genome-sequencing project. Because the vast majority of genome-sequencing projects are registered in the GOLD database [35], only those were considered. Species names were extracted from the GOLD database fields “Organism Name”, “Species” and “NCBI Project Name”; strain (deposit) names were extracted from these fields as well as from “Strain” and “Culture Collection”. To resolve synonyms between species names taxonomic information was collected from the LPSN website [36]. LPSN, which uses a nomenclature compatible with LTP [32], also provides lists of at least some of the deposits of the type strains of each species. These lists were augmented by searches in Straininfo [37].

The collected GOLD records and the taxonomic database were then compared as follows. A record was assigned the status “species not found” if none of the species names in the record were found in the taxonomic database. The status “strains not found” was assigned if at least one of the species names in the record was found in the taxonomic database, but none of the names of the strains from this record (original strain name or name of a deposits in a culture collection) were found in the type-strain list for this species in the taxonomic database. If both species name and according strain name synonyms were found, either the status “found-incomplete” or “found-complete” was used, depending on the project status as stated in the record. Entries with a “species not found” or “strains not found” status were considered as potential candidates for genome sequencing. The other type strains were not considered because their genome sequences were apparently already in progress or even finished. Because an initial screening revealed that misspelled taxon names play a minor role in GOLD, we used exact string matches to identify species names. Assigning strain names was also based on exact matching since strain names deemed too short for allowing partial matches only. We considered it beneficial, however, to relax this rule in three ways: (i) case-insensitive matching; (ii) equivalence of strain names that only differed by a “T” in the last position (which is often appended to indicate a type strain); and (iii) equivalence of strain names that only differed by characters other than letters, digits and underscores.

### Post-processing of the initial ranking

The 1,000 target strains for the main GEBA project were selected from the 8,029 ranked strains as follows. First, for obvious reasons, strains with genome projects registered in GOLD were removed. Second, strains not available in the DSMZ collection were removed. As not only the immediate accessibility of cryopreserved material, but also the generation of a sufficient amount of cell mass and the subsequent extraction of ultra-pure gDNA was necessary, it was deemed practical to postpone inaccessible strains to later phases of the project [10]. For the same reason, a small number of strains available in the holdings of the DSMZ but known as extremely challenging to cultivate (“fastidious”), were also disregarded in this phase of the project. This crucially necessary post-processing was eased considerably by the independence of the ranking of the selection of organisms.

### Target selection for genome sequencing within the *Roseobacter* clade

The *Roseobacter* clade is a major lineage within the *Rhodobacteraceae* (*Alphaproteobacteria*) [17,19]. At the time of target selection (spring 2011) it included about 95 species [36]. The clade is of interest mainly because of its important role in marine environments, where its members form one of the most abundant and successful groups of non-obligately phototrophic prokaryotes [18,38]. For a phylogenomic assessment of the group a suitable selection of organisms has to be obtained.

A phylogenetic tree including a total of 99 species was inferred from 1,366 aligned characters [39,40] of the 16S rRNA gene sequence under the maximum likelihood criterion [29,41,42]. For rooting, the genus *Labrenzia* (which belongs to the family *Rhodobacteraceae*, but not to the clade) was included but ignored when calculating the scores. (One of the advantages of these methods is that the ranking of the ingroup scores is independent of the ranking of the outgroup scores.)

## Results

### Interrelationships of phylogeny-based indexes for target selection

Table 2 show the correlations between the two measures, bRPD and uRPD, the heights in the tree of each leaf, and the number of nodes between the root and each leaf, and the residuals of the regression conducted with the latter two factors as the dependent and independent variable, respectively. Whereas bRPD and uRPD were highly correlated, their correlation with the number of nodes was moderately strong and negative. Since the number of nodes between a leaf and the root is inversely proportional to the size of its topological isolation, this result indicates that both measures comply with the third design goal (to positively weight topological isolation). The tree height of the leaves, i.e. the sum of the lengths of all branches connecting a leaf with the root node of the tree, was slightly but significantly (α = 1.0e^-40^) negatively correlated with both bRPD and uRPD. Even though this behavior is in obvious conflict with the second design goal, the correlation between tree height and number of nodes between root and leaf must be considered (Table 2). If the effect of the number of nodes is corrected for by replacing the tree height with the residuals from a regression with the number of nodes as explanatory variable, the correlation to the bRPD and uRPD becomes moderately strong and positive.

**Table 2:** Correlations between the balanced (bRPD) and the unbalanced (uRPD) variant of the score for each leaf (“ Height”).

	**bRPD**	**uRPD**	**Height**	**# nodes**	**Residual**
**bRPD**		<1.0e^-40^	<1.0e^-40^	<1.0e^-40^	<1.0e^-40^
**uRPD**	0.8004		<1.0e^-40^	<1.0e^-40^	<1.0e^-40^
**Height**	-0.0672	-0.0972		<1.0e^-40^	<1.0e^-40^
**# nodes**	-0.3140	-0.3329	0.5798		0.1546
**Residual**	0.2907	0.2591	0.4372	0.0107	

**Figure 2:**
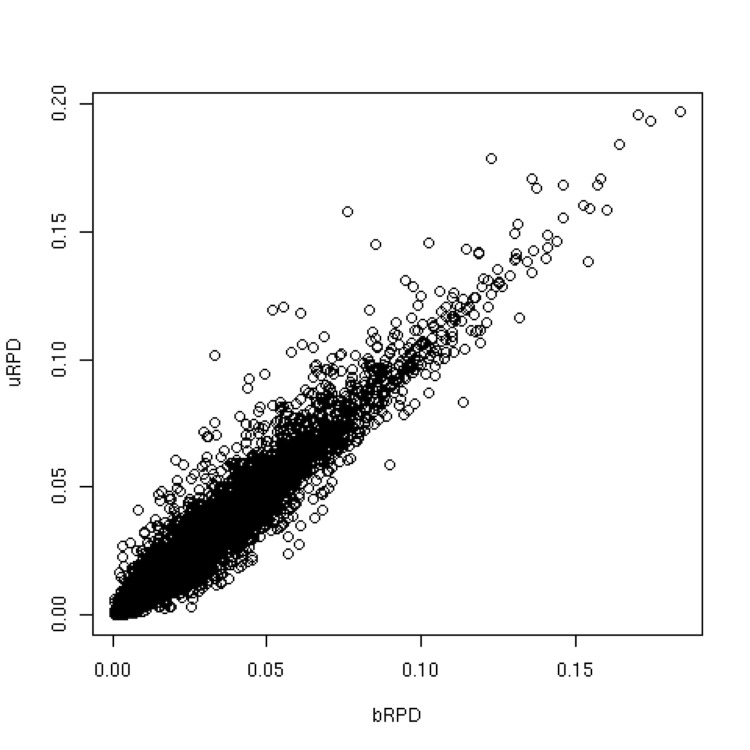
Scatterplot showing the relationship between the two examined variants of the phylogenetic scoring, bRPD (x-axis) and uRPD (y-axis). In addition to the fact that the overall correlation between the two measures is high (see also Table 2), it is obvious that the distribution of both variants is highly right-skewed; that is, few strains with high scores are accompanied by a bulk of strains which contribute only little to the overall sum of the scores.

Based on these results, we concluded that both measures comply with design goals (i) and (ii), but finally preferred bRPD because it showed more well-balanced correlations with the indicator of topological isolation on the one hand and the independent effect of the branch lengths on the other hand than uRPD. But the differences between both measures were not pronounced, particularly regarding the top-scoring species; in addition to Table 2, this is shown in the scatter plot in Figure 2 and in Table 3.

**Table 3:** Selection results for the 20 LTP strains with the highest bRPD scores.

**Species/subspecies**	**16S rRNA accession [**32**]**	**Phylum [**36**]**	**bRPD**	**uRPD**	**Category**
*Caldisericum exile*	AB428365	*Caldiserica*	0.1841	0.1968	Targeted elsewhere
*Asteroleplasma anaerobium*	M22351	*Tenericutes*	0.1747	0.1936	Not at DSMZ
*Phycisphaera mikurensis*	AB447464	*Planctomycetes*	0.1703	0.1960	Targeted elsewhere
*Ktedonobacter racemifer*	AM180156	*Chloroflexi*	0.1646	0.1839	Targeted in GEBA pilot project
*Fibrobacter succinogenes subsp. succinogenes*	AJ496032	*Fibrobacteres*	0.1604	0.1586	Completed elsewhere
*Exilispira thermophila*	AB364473	*Spirochaetes*	0.1581	0.1709	Not at DSMZ
*Bdellovibrio bacteriovorus*	AJ292759	*Proteobacteria*	0.1575	0.1684	Completed elsewhere
*Flexibacter litoralis*	AB078056	*Bacteroidetes*	0.1547	0.1589	Targeted in GEBA pilot project
*Lactobacillus catenaformis*	AJ621549	*Firmicutes*	0.1541	0.1382	Selected
*Lentisphaera araneosa*	AY390428	*Lentisphaerae*	0.1526	0.1601	Targeted elsewhere
*Gemmatimonas aurantiaca*	AB072735	*Gemmatimonadetes*	0.1461	0.1555	Completed elsewhere
*Dehalogenimonas lykanthroporepellens*	EU679419	*Chloroflexi*	0.1460	0.1681	Completed elsewhere
*Zavarzinella formosa*	AM162406	*Planctomycetes*	0.1440	0.1466	Selected
*Gemmata obscuriglobus*	X56305	*Planctomycetes*	0.1411	0.1437	Targeted elsewhere
*Victivallis vadensis*	AY049713	*Lentisphaerae*	0.1410	0.1485	Targeted elsewhere
*Peredibacter starrii*	AF084852	*Proteobacteria*	0.1406	0.1395	In progress elsewhere
*Thermodesulfobium narugense*	AB077817	*Firmicutes*	0.1377	0.1670	Targeted in GEBA pilot project
*Nitrospira moscoviensis*	X82558	*Nitrospira*	0.1363	0.1424	In progress elsewhere
*Hydrogenobaculum acidophilum*	D16296	*Aquificae*	0.1360	0.1707	Postponed for technical reasons
*Fibrobacter intestinalis*	AJ496284	*Fibrobacteres*	0.1358	0.1341	Not at DSMZ

The column “Category” indicates whether or not the strain was selected for sequencing, and if not, whether this was due to the strain being already targeted in another genome-sequencing project or due to technical reasons, or whether the maximum of eight genome-sequencing projects had already been reached.

The number of nodes between the root and each leaf (“# nodes”) and the residuals of a linear regression with the number of nodes as explanatory and the height as dependent variable (“Residual”). These residuals represent the average impact of the branch lengths, independent of the number of branches that contribute to the height. The lower left triangle shows Kendall's correlation coefficients, the upper right triangle shows the corresponding p values.

### Selection of targets for genome sequencing

**Figure 3:**
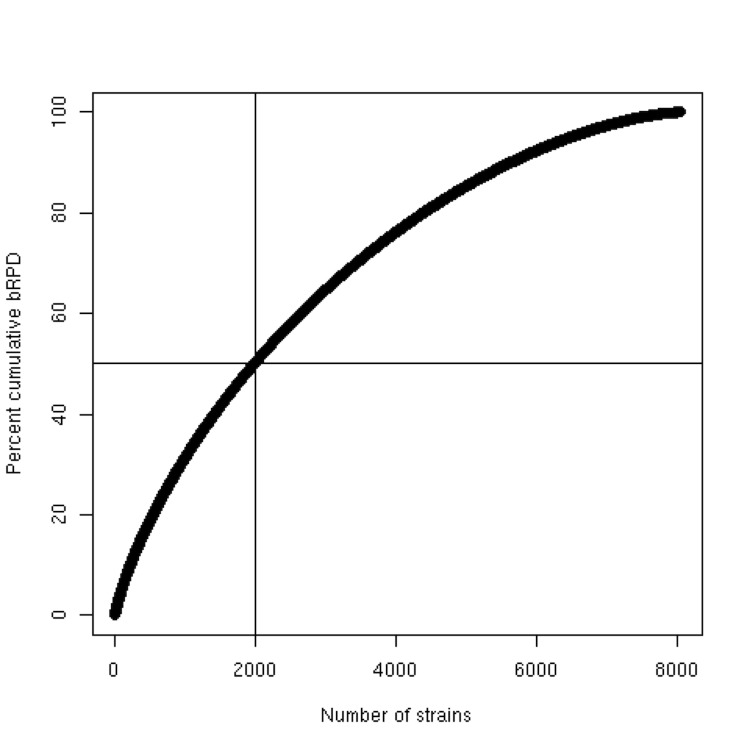
Saturation plot for the bRPD measure. X-axis, index of the decreasingly sorted bRPD values; y-axis, cumulative bRPD sum in percent. The right-skewed distribution of the bRPD values (see Figure 2) manifests itself in the fact that only about 2,000 strains (vertical line) are necessary to reach 50% of the overall phylogenetic diversity (horizontal line) as estimated using this measure.

In addition to the close correspondence between the two measures, Figure 2 demonstrates that the distribution of both bRPD and uRPD is strongly asymmetric, as comparatively few strains (close to upper right corner) display very high values compared to the bulk of the strains which show at most moderately high bRPD and uRPD measures (close to the lower left corner). This behavior is confirmed by Figure 3, which shows that 50% saturation regarding bRPD would already be obtained if only about 2,000 of the 8,029 strains were genome sequenced.

Using bRPD as primary selection criterion and matching the GOLD database in the current version during the GEBA phase I planning period (December 2010) resulted in the following numbers. Among the total of 8,029 strains, 453 had a “completed” genome-sequencing project, 38 a project “in progress”, and 766 a “targeted” project. Among the remaining strains lacking a genome-sequencing project registered in GOLD at the time being, 7 were *Cyanobacteria*, 970 were not contained in the holdings of the DSMZ, 36 had to be rejected for technical reasons, 685 were set aside as replacement strains in case any of the 1,000 targeted ones turned out to pose difficulties in sequencing. Finally, 4,074 strains with low scores or expected technical difficulties remained that were postponed and not considered for this phase of the project. Some of the strains not available at DSMZ were selected using the same procedure for potential targeting by the ATCC, Manassas, VA.

Table 3 shows the results for the 20 highest-scoring strains according to the bRPD. Apparently, strains from a considerable diversity of phyla are included in the list, and mainly from sparsely sampled phyla with accordingly high inter-species differences [32,36]. Only comparatively few strains had to be postponed or rejected because of their current unavailability or for technical reasons related to cultivation and gDNA extraction. Most of the strains that were not selected were known as targets of other genome projects (or the GEBA pilot project).

### Stability of the scoring

The comparison of the LTP release “LTPs106” with release “LTPs102” revealed that 7,991 of the INSDC 16S RNA accessions used in LTPs102 were still in use in the more recent dataset. The Kendall correlation between the bRPD values from both releases after restricting the dataset to the common accessions was 0.925; for uRPD, it was 0.917. Among the 1,000 accessions of the LTPs102 release with the highest bRPD score, 76 were not among the highest-scoring ones from LTPs106; if uRPD was used, this number amounted to 83. This result indicates an additional advantage of bRPD over uRPD.

### Suitable targets for genome sequencing within the *Roseobacter* clade

**Figure 4:**
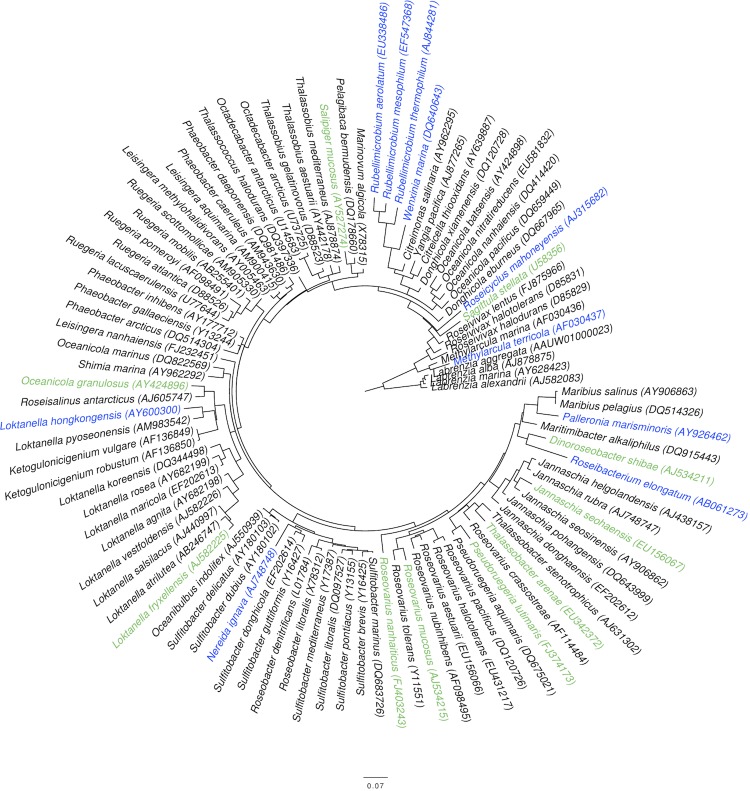
Phylogenetic tree of the members of the *Roseobacter* clade (known at the time of target selection) rooted with *Labrenzia* spp. The branches are scaled in terms of the expected number of substitutions per site (see size bar). Bootstrap support values [43] were calculated but have been omitted for clarity because they are not relevant to the scoring. The organisms with the ten highest bRPD scores are marked in blue. The organisms with the ten next highest bRPD scores (ranks 11 to 20) are marked in green.

The phylogenetic tree used for target selection within the *Roseobacter* clade is shown in Figure 4, whereas Supplementary Table 1 includes the scores for the species. As expected, the scoring preferred species situated in isolated positions (e.g., *Methylarcula terricola*) and/or at long branches (e.g., *Rubellimicrobium* spp.). Eight species were selected for genome sequencing (see Supplementary Table 1 for details), among them was *Roseibacterium elongatum,* the one with the overall highest score.

## Discussion

As shown above, the scoring algorithm complies with the four design goals and is also easy to comprehend and implement. Even though written in a scripting language, the algorithm already runs reasonably fast (few seconds for the LTPs104 tree on a modern workstation), particularly if compared to the running time needed for inferring a maximum-likelihood tree for so many leaves. For several reasons listed above, bRPD seems to be preferable over uRPD, even though the differences are not dramatic (Figure 2). The correlation between bRPD values from distinct LTP releases (if reduced to the common 16S rRNA accessions) was even higher, indicating a sufficient stability of the scoring.

Both measures yielded a strongly asymmetric (right-skewed) distribution of the scores (Figure 2). This is expected, given the usual asymmetry of phylogenetic trees, i.e. their tendency to contain sister clades of highly unequal sizes [28]. Also, evolution seldom occurs according to a molecular clock [28], thus allowing for higher variability regarding the branch lengths. In practice, it means that a large proportion of the overall phylogenetic diversity can be covered with comparatively few well selected organisms (Figure 3).

It cannot entirely be avoided that interesting species are missing in the tree used for target selection. For instance, at the time of writing the *Roseobacter* clade contained 117 species [36], 22 more than when the genomes were selected for sequencing (Figure 4, Supplementary Table 1). Many interesting organisms, even if discovered in environmental samples, might not be cultivable with current techniques. The examples from real-world genome-sequencing projects shown here clearly indicate that this is often the limiting factor (Table 3, Supplementary Table 1). Whether or not such organisms can be targeted in the close future using techniques such as single-cell genome sequencing [44,45] remains to be seen.

The species with high scores were mainly from a considerable diversity of sparsely sampled phyla with accordingly high inter-species differences (Table 3), indicating that the suggested index indeed addresses phylogenetic diversity. This is supported by the *Roseobacter*-clade example (Figure 4, Supplementary Table 1), where species rather isolated from their phylogenetic neighbors were primarily targeted. It is also not surprising that a number of species that have already been selected for the GEBA pilot project appeared among the top-scorers, even though the novel scoring is not equivalent to the previously used one. Thus, whether or not the algorithm introduced here will yield a similar or even higher degree of novel protein families in the genomes targeted in GEBA phase I [10] is a question that can only be solved once these genomes have been sequenced. According to the considerations listed above, the new scoring is quite promising, however.

It should not be overlooked that the scoring can be affected by a number of artifacts because of its dependence on the underlying phylogenetic tree and the annotation of its leaves. For instance, LTP versions have sometimes selected the wrong sequence as, e.g., in the case of the type strain of *Weeksella virosa* [46]. But compared to the overall number of strains (Figure 3) these problems appear to be rare. Moreover, to avoid picking the wrong organisms in the GEBA project the 16S rRNA gene of each strain is resequenced after gDNA extraction, and the strain is put back if the sequence does not match database sequences annotated as being obtained from the same strain. Using a phylogenetic tree of some organisms instead of their taxonomic classification avoids a number of potential artifacts in taxon selection. Even though it has only slowly been appreciated by taxonomists after Darwin, the sole possible goal of a taxonomic classification is to summarize the genealogy of the organisms [3,4]. For this reason, a taxonomic classification always contains less information than the empirical estimate of the phylogeny from which it was derived. But frequently classifications cannot even pretend to summarize the respective underlying genealogies because the classifications include non-monophyletic groups [3,4,47,48]. Current microbial classification contains a number of such taxa (e.g., *Bacillus* [15], *Desulfotomaculum* [49], *Planctomyces* [43], *Spirochaeta* [16] and *Xanthobacteraceae* [50]).

Some of the problematic parts of the classification are due to missing phylogenetic analyses in the original description (e.g., [15]), often because suitable character data or inference methods were simply lacking at the time when the taxon was described (e.g., [16]). But in other cases, such problematic taxa have been created due to conceptual shortcomings. For instance, the genus *Schlesneria* was introduced in a study [51] in which a tree was depicted that clearly showed that the placement of the new taxon causes another genus, *Planctomyces*, to become paraphyletic [43] (see [52] for algorithmically straightforward, character-independent definitions of the terms “monophyletic”, “paraphyletic” and “polyphyletic”). Clearly, such discrepancies are not due to preferring phenotypic traits (used as “diagnostic” characters) over 16S rRNA gene results because diagnostic characters are not necessarily synapomorphies. But only synapomorphies (or phylogenetic trees, of course [52]) can justify monophyletic groups [3,53]. For instance, it is easy to outline the diagnostic characters of reptiles that separate them from either mammals or birds, but nevertheless reptiles are the classical example of a paraphyletic group [3].

Finally, even if a classification would only contain monophyletic groups, a prevailing major obstacle against using it for target selection was that ranks in Linnean hierarchies cannot quantitatively be compared, because they might reflect largely distinct levels of character divergence [3]. Thus, targeting, e.g., one species per genus might not be a wise choice, even if all genera were monophyletic. Only for the species rank, microbial taxonomy has firmly established a criterion related to character divergence, namely the DNA-DNA hybridization (DDH), traditionally conducted in the wet lab [6] but more recently using genome-sequence based, digital replacements [47]. DDH, however, is a similarity method, whereas more similar organisms are not necessarily more closely related [3,28,48,53].A further problem with the approach to generate one genome per taxon (of a chosen taxonomic rank) is that the number of genomes to be sequenced would not depend on the available project resources but on the number of taxa. Neither a ranking within nor between those taxa would be provided. The same difficulty would arise if non-hierarchical sequence clustering was used, followed by selecting one organism per cluster, even though here the number of clusters could be chosen (using, e.g., K-means partitioning [54]) and thus adapted to the project's needs. But in contrast to the suggested phylogeny-based scoring, no continuous ranking would be provided, and re-clustering would be necessary after each change in the number of target genomes. Using trees with branch lengths for target selection thus seems to be the best choice, and the ease with which scoring systems such as the one described here can be inferred from phylogenies renders such methods rather promising.
